# Integration of experimental data with model prediction and simulation reveals how Mettl15-Mettl17 modulates pre-mitoribosome.

**DOI:** 10.1063/4.0001094

**Published:** 2025-10-27

**Authors:** Alexey Amunts

**Affiliations:** Munster University, Munster, Germany, Germany

## Abstract

We used an integrative approach combining cryo-EM data, molecular dynamics simulations, and computational modeling to uncover intermediate states and propose a sequential assembly mechanism of the mitoribosome orchestrated by the methyltransferases Mettl15 and Mettl17. Our analysis identified previously unassigned elements in the cryo-EM map of T. brucei mitoribosomal small subunit precursor, including homologs of assembly factors RbfA and Mettl15 tightly associated with Mettl17. Phylogenetic studies revealed the conservation of the Mettl15-Mettl17 heterodimer across eukaryotic groups. Using AlphaFold and molecular dynamics simulations, we modeled the human pre-mitoribosome and demonstrated the dynamic role of Mettl17 in recruiting Mettl15 and facilitating rRNA maturation through conformational rearrangements. Our results suggest that Mettl17 primarily acts as a structural organizer rather than an enzymatic methyltransferase. Finally, we combined our analysis with previously obtained structural insights into a coherent series of sequential assembly steps that lead to the catalytic mitoribosome. This work highlights how combining molecular dynamics with template-based modeling can reveal transient states, offering a powerful tool for studying complex macromolecular systems where experimental data are limited. This broadly applicable methodology can ultimately help reveal protein functions and more complete biogenesis pathways in other metabolic processes.

